# Induction gemcitabine in standard dose or prolonged low-dose with cisplatin followed by concurrent radiochemotherapy in locally advanced non-small cell lung cancer: a randomized phase II clinical trial

**DOI:** 10.2478/raon-2014-0026

**Published:** 2014-11-05

**Authors:** Martina Vrankar, Matjaz Zwitter, Tanja Bavcar, Ana Milic, Viljem Kovac

**Affiliations:** 1 Institute of Oncology Ljubljana, Ljubljana, Slovenia; 2 Faculty of Medicine, University of Maribor, Slovenia; 3 Clinical Radiology Institute, University Medical Centre Ljubljana, Slovenia

**Keywords:** induction chemotherapy, gemcitabine, non-small cell lung cancer, radiation therapy, concurrent chemoradiation, randomized clinical trial

## Abstract

**Background:**

The optimal combination of chemotherapy with radiation therapy for treatment locally advanced non-small cell lung cancer (NSCLC) remains an open issue. This randomized phase II study compared gemcitabine in two different schedules and cisplatin - as induction chemotherapy, followed by radiation therapy concurrent with cisplatin and etoposid.

**Patients and methods.:**

Eligible patients had microscopically confirmed inoperable non-metastatic non-small cell lung cancer; fulfilled the standard criteria for platin-based chemotherapy; and signed informed consent. Patients were treated with 3 cycles of induction chemotherapy with gemcitabine and cisplatin. Two different aplications of gemcitabine were compared: patients in arm A received gemcitabine at 1250 mg/m^2^ in a standard half hour i.v. infusion on days 1 and 8; patients in arm B received gemcitabine at 250 mg/m^2^ in prolonged 6-hours i.v. infusion on days 1 and 8. In both arms, cisplatin 75 mg/m^2^ on day 2 was administered. All patients continued treatment with radiation therapy with 60–66 Gy concurrent with cisplatin 50 mg/m^2^ on days 1, 8, 29 and 36 and etoposid 50 mg/m^2^ on days 1–5 and 29–33. The primary endpoint was response rate (RR) after induction chemotherapy; secondary endpoints were toxicity, progression-free survival (PFS) and overall survival (OS).

**Results:**

From September 2005 to November 2010, 106 patients were recruited to this study. No statistically signifficant differences were found in RR after induction chemotherapy between the two arms (48.1% and 57.4%, p = 0.34). Toxicity profile was comparable and mild with grade 3/4 neutropenia as primary toxicity in both arms. One patient in arm B suffered from acute peripheral ischemia grade 4 and an amputation of lower limb was needed. With a median follow-up of 69.3 months, progression-free survival and median survival in arm A were 15.7 and 24.8 months compared to 18.9 and 28.6 months in arm B. The figures for 1- and 3-year overall survival were 73.1% and 30.8% in arm A, and 81.5 % and 44.4% in arm B, respectively.

**Conclusions:**

Among the two cisplatin-based doublets of induction chemotherapy for inoperable NSCLC, both schedules of gemcitabine have a comparable toxicity profile. Figures for RR, PFS and OS are among the best reported in current literature. While there is a trend towards better efficacy of the treament with prolonged infusion of gemcitabine, the difference between the two arms did not reach statistical significance.

## Introduction

Lung cancer remains the most common cause of cancer related deaths in the world. In Europe, approximately 410.000 lung cancer patients were diagnosed and 353.000 individuals were estimated to die from lung cancer in 2012.^1^,^2^ Non-small cell lung cancer (NSCLC) accounts for approximately 85% of all primary lung cancers, of whom about one fourth have locally advanced disease.^3^ The standard treatment for patients with surgically unresectable, locally advanced NSCLC includes concurrent radiation therapy and chemotherapy.^4^,^5^ According to a meta-analysis by Auperin *et al.*, concurrent regimens are superior to sequential ones in terms of locoregional control and overal survival.^5^,^6^ Long-term survival rates with these approaches are only in the order of 15%. Considering the fact that with concurrent schedules, the chemotherapeutic agents enhance the tumor′s radiosensitivity and thus improve the local control but have little if any systemic effect, improvement in overall survival can be achieved through better control of distant micrometastases.

Applied either sequentially or in a concurrent schedule, platinum-based chemotherapy with radical radiation therapy is the standard of care for locally advanced NSCLC as well as for small cell lung cancer (SCLC).^7^,^8^ However, the optimal drugs, schedule, sequence and doses of chemotherapy have not been adequately defined.

Gemcitabine is among the standard drugs for the treatment of a variety of tumors, including NSCLC.^9^,^10^ For the usual 30-minute infusion (dose rate 40–60 mg/m^2^/min), the maximum tolerated dose (MTD) is 1500 mg/m^2^ or even higher.^11^,^12^ With infusion lasting for 3, 6 or 24 hours, MTD significantlly falls to 450, 250 and 180 mg/m^2^, respectively.^13^–^15^ This phenomenon can be explained by saturation of deoxycytidin kinase, an encyme needed for conversion of gemcitabine into its active form gemcitabine-triphosphate. After a short infusion of a relatively high dose gemcitabine, most of the drug remains unmetabolized. By contrast, prolonged infusion leads to higher intracellular concentration of the active metabolite.^16^ Consequently, a lower dose is needed for a comparable activity.

Several phase I and II clinical trials have shown significant antitumor activity of gemcitabine in low-dose in long infusion. The spectrum of diseases includes cancers of the lung, breast, pancreas, gallbladder, bladder, sarcomas, mesotheliomas, refractory leukemias, and refractory Hodgkin′s disease.^14^,^17^ Regarding lung cancer, our group reported favorable experience with gemcitabine in long infusion in combination with cisplatin for metastatic NSCLC.^9^,^10^,^18^

After a favorable experience with gemcitabine at a low-dose in prolonged infusion for advanced NSCLC, we here present a phase II randomised trial of induction chemotherapy comparing gemcitabine in two different schedules of application in combination with cisplatin followed by concurrent radiochemotherapy.

## Patients and methods

### Eligibility criteria

Patients with medically or surgically inoperable citologically or histologically confirmed NSCLC or locall reccurence after previous surgical treatment were eligible for the trial. Patients were required to be 18 years of age or older, have a performance status (PS) of 0–1 based on the Eastern Cooperative Oncology Group, with no evidence of metastatic disease, with no previous chemotherapy or radiation therapy for NSCLC, with no other malignant disease for last three years (except basal cell carcinoma of the skin, carcinoma *in situ* of the cervix or carcinoma of larynx T1N0M0) and have adequate hematological, kidney and liver function. Patients were ineligible if they had malignant pleural or pericardial effusions, evidence of manifest cardial or nevrologic disease or evidence of active infection. All patients were discussed on multidisciplinary thoracic oncology tumor board and considered inoperable due to tumor extent, limited pulmonary function or other comorbidity.

Radiological assessment included chest x-ray, CT scan of the torax, abdomen and brain and technetium-99 bone scan, or FDG-PET-CT examination when available. All studies, including a complete medical history and physical examination, were completed within 2 weeks before study enrollment.

All patients were fully informed and signed a consent to participate in the trial.

The protocol was approved by the Institutional Review Board (Institute of Oncology Ljubljana) and by the National Committee for Medical Ethics, Ministry of Health, Republic of Slovenia.

### Treatment

Patients were randomly assigned to one of the two treatment arms. All patients were treated with three 21-day cycles of induction chemotherapy. We compared two different methods of applications and dosage of gemcitabine, administered as induction chemotherapy: patients in arm A received 1250 mg/m^2^ in standard half hour i.v. infusion on days 1 and 8; patients in arm B received gemcitabine 250 mg/m^2^ in prolonged 6-hours i.v.infusion on days 1 and 8. In the both arms, cisplatin 75 mg/m^2^ on day 2 intravenously was administered.

Within 13–22 days after the last aplication of chemotherapy, all patients continued treatment with radiation therapy concurrent with cisplatin 50 mg/m^2^ on days 1, 8, 29 and 36 and etoposide 50 mg/m^2^ on days 1–5 and 29–33.

Radiation therapy was administered with a linear accelerator photon beam of 5–10 MV in 2 Gy fractions 5 times weekly to a total dose of 60–66 Gy. Three-dimensional CT-based conformal radiation therapy was used and treatment planning was based on CT scans obtained under normal quiet breathing. The tumor volumes: gross tumor volume (GTV), clinical target volume (CTV), planning target volume (PTV) and organs at risk were delineated. GTV encompassed the primary tumor before chemotherapy and involved lymph nodes determined from diagnostic CT or FDG-PET-CT. CTV was defined as the GTV plus the margin for microscopic extension of the tumor (5 mm) and PTV was defined as CTV plus an additional margin for organ and patient movement (10–15 mm). No elective nodal volumes were included. The dose was prescribed to the isocenter. Tissue heterogeneity correction was performed and the superposition dose calculation algorithm was used. Normal tissue tolerance criteria for the spinal cord, esophagus and lung were specified accordance to Emami normal tissue tolerance tables. Dosimetric parameters were generated from the dose-volume histogram (DVH).

Toxicities were assessed according to Common Terminology Criteria for Adverse Events (CTCAE) version 3.0. The protocol contained guidance for adjustments to adverse events. However, induction chemotherapy should follow schedule with dose reduction or omiting drug application as indicated in protocol. Radiotherapy interruptions or delay were permited for grade 3/4 adverse events.

### Treatment assessment

After induction chemotherapy, response of the tumor was assessed by comparing the pre-treatment CT scan with the CT scan of the torax before starting radiation therapy. The response was evaluated according to Response Evaluation Criteria in Solid Tumor (RECIST) criteria version 1.0. In addition, volumetric measurement of tumor on CT scans before and after induction chemotherapy was performed. All three dimensions were measured by a radiologist blinded regarding treatment allocation. The volume was calculated as cuboid shape for each tumor before and after induction chemotherapy, and the percent of response was calculated.

After completion of treatment, patients were evaluated at 6 weeks and every third month thereafter. In addition to clinical exam, chest x-ray and blood tests which were done during every follow-up visit, CT scan of the torax was performed at 5 months after treatment and every year thereafter or earlier if clinically indicated.

### Statistical analysis

The primary endpoint of this prospective randomized open-label, phase II trial, was response rate (RR) after induction chemotherapy, and secondary endpoints included progression free survival (PFS), overall survival (OS) and safety profile.

PFS was defined as the time from the beginning of treatment to disease progresion or death. OS was calculated as the time from the start of the treatment to death from any cause. Censoring was defined as the time from the beginning of treatment to the last contact with the patient and for alive patients, as the time from the beginning of treatment to the end of follow-up (October 2013).

Overall and progression-free survival curves were estmated by using Kaplan-Meier method and log-rank test. Chi-square test was used to compare distribution of discrete variable values between the two arms. Mann-Whitney U test was used to compare continuous variables. Z-test for the equality between two proportions was used to evaluate the difference between proportions of patients between arms. A p-value less than 0.05 was considered statistically significant.

## Results

### Patient characteristics

Between September 2005 and November 2010, a total of 107 patients were randomly assigned to the arm A (53 patients) or the arm B (54 patients). One patient in group A was ineligible due to grade 3 cardial failure immediately after starting the infusion of first application of chemotherapy, so she continued treatment with radiation therapy only. Patient demographics and disease characteristics are listed in Table 1.

Most patient had surgically inoperable tumor in stages IIIA and IIIB (94% arm A and 96% arm B). Four patients were inoperable due to poor pulmonary function and 1 patient refused surgical treatment. Most patients had no previous treatment (92% arm A and 82% arm B). Thirteen patients were referred for radiochemotherapy after exploratory thoracotomy and 1 patient had a reccurence 1 year after surgery. The most predominant histological subtype was squamous cell carcinoma (67% arm A and 50% arm B). The differences between the two arms were not statistically significant.

### Treatment administered

The treatment delivery for 106 patients in both arms is listed in Table 2. A total of 28 patients (53.8%) in arm A and 24 (44.4%) in arm B received all 3 planned cycles of induction chemotherapy. One patient in arm A and 3 patients in arm B received only one cycle of induction chemotherapy. The dose intensity, measured as mean value of percentage of drug administered was for cisplatin and gemcitabine 87.7% and 89.6% for arm A and 87.2% and 84.7% for arm B, respectively. After induction chemotherapy one patient in arm B undervent surgery and pulmectomy was performed.

In two patients in arm A radiation therapy was initiated with paliative intent due to extent of the tumor. Radical radiation therapy with doses of ≥ 56 Gy was completed in 48 patients (92.4%) in arm A and in 53 patients (98.1%) in arm B. Fifteen patients (28.8%) in arm A and 19 patients (35.2%) in arm B received 2 planned cycles of concurrent chemotherapy, and to 11 patients in each group no concurrent chemotherapy was given. Main reasons for omitting concurrent chemotherapy were hematological toxicity and esophagitis.

### Toxicity

Treatment-related acute toxicities of induction chemotherapy were mild and are listed in Table 3. Grade 3 or 4 adverse events were comparable in both arms. No one had febrile neutropenia or grade 3 or more acute kidney injury. Alopecia was more frequent in arm B (15.4% *vs*. 42.6%, p = 0.004). After second cycle of induction chemotherapy, one patient in arm B suffered from grade 4 acute peripheral ischemia leading to amputation.

Treatment-related acute toxicities of concurrent radiochemotherapy are listed in Table 4. There were statistically significantly higher rates of grade 3/4 anemia and thrombocytopenia in arm A with 7.7% (p = 0.04) and 9.6% (p = 0.02) compared with arm B with no grade 3/4 anemia and thrombocytopenia. Two patients in arm A suffered from sepsis after 20 Gy and 22 Gy of radiaton therapy and first cycle of concurrent chemotherapy. Afterwards, the first patient never continued treatment of lung cancer due to cardial failure leading to his death 3 months later. The second patient developed grade 3 infective pericarditis and glomerulonephritis. During treatment of these complications the brain metastases developed, leading to his death two months after interruption of chest irradiation.

Two patients in arm A died from pneumonia short time after completion of treatment – after 2 weeks and 2 months. In both patients pneumonia was associated with radiation pneumonitis. However, the dose of delivered irradiation was within the restrictions for lung tissue in both cases. An autopsy in one patient revealed aspergiloma and necrosis with some malignant cells at the site of the tumor.

### Response and survival

The primary endpoint of the study was RR after induction chemotherapy. All 106 patients in both arms were analyzed for response according RECIST criteria (Table 5). No complete responses were seen. Partial response and stable disease were achieved in 25 patients (48.1%) and 27 patients (51.9%) for arm A, and in 31 patients (57.4%) and 22 patients (40.7%) for arm B, respectively. One patient in arm B had progressive disease.

Five month after completion of treatment, 82 patients were evaluable for response according to RECIST. RR was observed in 32 patients (84%) in arm A and in 33 patients (75%) in arm B.

**FIGURE 1.**
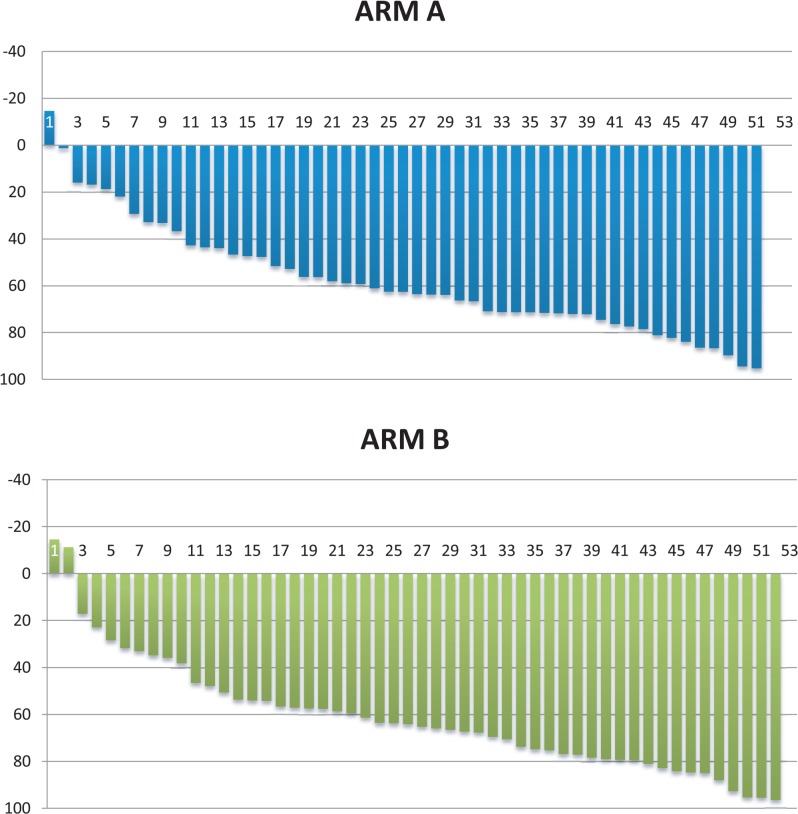
Waterfall plot for reduction of the tumor volume after induction chemotherapy for arm A and arm B.

Regarding volumetric measurements, we observed median reduction of the tumor volume for 62.2% in arm A and 64.7% in arm B (p = 0.41) (Figure 1).

**FIGURE 2.**
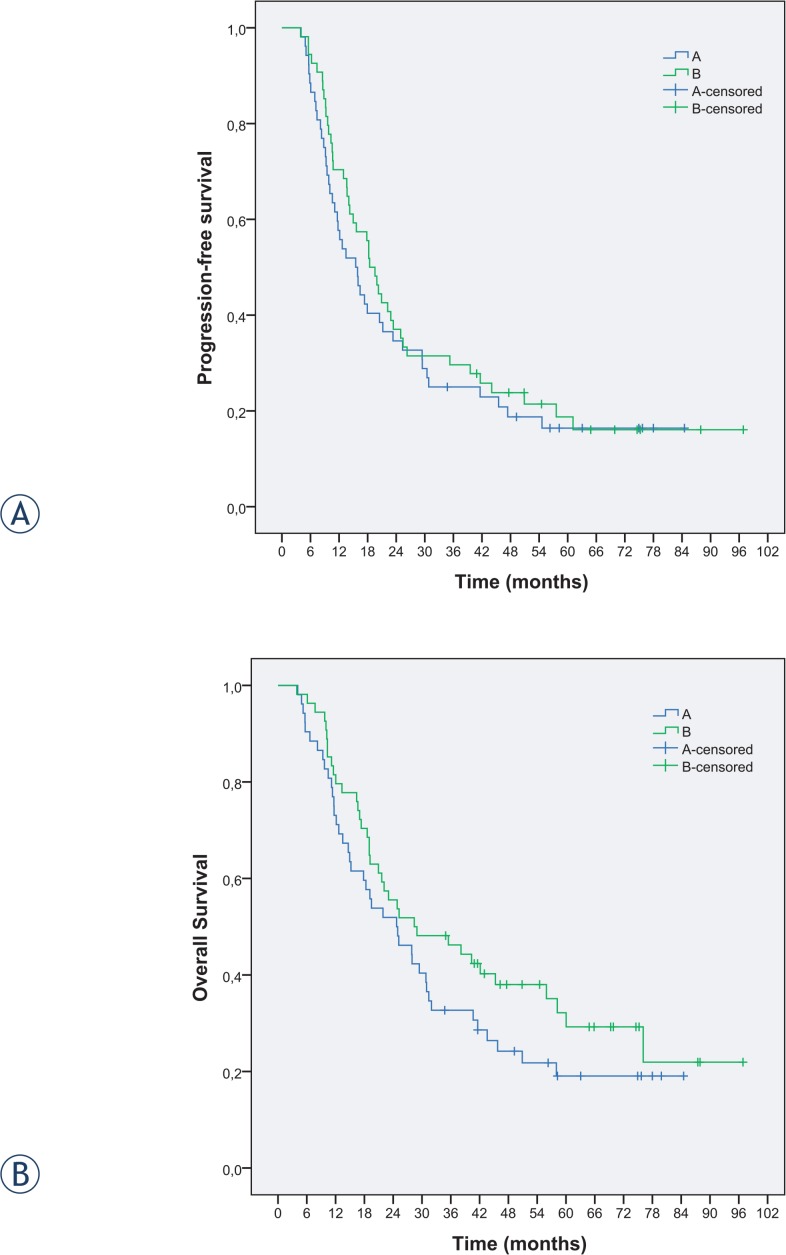
**(A)** Progression-free survival and **(B)** overall survival for the two arms.

The PFS and OS data are shown in Figure 2. No satatistically significant difference in PFS and OS was recognized between the two arms. Median follow-up time of surviving patients was 69.3 months (range 60–72 months). Median PFS was 15.7 months in arm A and 18.9 months in arm B (p = 0.24). The OS in arm A was 24.8 months compared to 28.6 months in arm B (p = 0.18). The OS rates at 1, 2, 3 and 5 years were 73.1%, 51.9%, 32.7% and 19.1% in arm A and 81.5%, 55.6%, 46.2% and 32.2% in arm B, respectively.

Three months after completion of chemo-radiotherapy, one patient in arm B undervent pulmectomy and histologically complete response was confirmed. This patient is still alive with no sign of progression.

Three and a half months after completion of treatment one patient in arm A died from pulmonary embolism, confirmed by autopsy.

One patient died from acute lymphatic leukemia two years after the treatment without progresion of lung cancer.

Two patients were affected with second primary cancer, one with new lung cancer six years after first treatment and one with carcinoma of oral cavity also six years after treatment of lung cancer. Both were treated with radiochemotherapy and are still alive.

At the time of last evaluation in October 2013, 28 patients were alive and 19 without disease, 11 and 9 in arm A and 17 and 10 in arm B, respectively.

The sites of initial relapse among 37 patients in arm A were locoregional in 18 patients (48.6%), distant in 12 patients (32.5%) and both locoregional and distant in 7 patients (18.9%), and among 39 patients in arm B locoregional in 21 patients (53.9%), distant in 10 patients (25.6%) and both in 8 patients (20.5%).

## Discussion

This prospective randomised phase II trial resulted in the median survival of 24.8 months in arm A and 28.6 months in arm B. Three and 5-year estimated survival rates of 32.7% and 19.1% in arm A and 46.2% and 32.2% in arm B suggest an improved median survival and overall survival in group B compared to group A; however, the difference was not statistically significant. It should be noted that a slight imbalance existed between the two arms. A higher proportion of patients in the arm B had adenocarcinoma histology, was stage III A and previously had explorative thoracotomy. In addition, more patients in arm B received > 56 Gy of radiotherapy, although these differences were not statistically significant.

The primary endpoint of RR after induction chemotherapy revealed no difference between the two groups by RECIST criteria or by volumetric measurement. We conducted volumetric measurement to precisely identifiy differences in the tumor reduction in each groups, however the results are comparable in both groups.

Results of RR after induction chemotherapy in our trial were consistent with some recently published reports. PR and SD in our series were 48.1% and 51.9% in arm A and 57.4% and 40.7% in arm B, respectively. Only one patient in arm B (1.9%) had progressive disease. In the study by Schallier *et al*., with 64 patients constituting the study population, 55% PR was obtained after three cycles of triplet induction chemotherapy regimen of paclitaxel, carboplatin and gemcitabine (PACCAGE).^19^ In the study by Hirsh *et al*. of 41 assessable patients who were treated with induction two cycles of carboplatin and gemcitabine 73.1% achieved PR and 24.4% SD.^20^ Other publications from recent years showed lower PR and SD of 37% and 50% after two cycles of induction cisplatin and oral vinorelbine^21^, PR and SD of 36% and 52% after two cycles of induction gemcitabine and vinorelbine^22^, and PR and SD of 32.1% and 44.6% after two cycles of induction cisplatin and docetaxel.^23^

Compliance to induction chemotherapy in our series was good considering the dose-intensity, with 87% of administered cisplatin in both arms and 89% and 84% of gemcitabine administered in arm A and arm B. However, 53.8% and 44.4% of patients in arm A and arm B received full three cycles of induction chemotherapy. These numbers were quite low but it should be stressed that the schedule of chemotherapy was fixed and aplications of drugs were not delayed but omitted in the case of toxic side effects.

In the concurrent radiochemotherapy the primary objective was completion of radiotherapy without interruption. Dose intensity for arm A and arm B was 61.7% and 67.8% for cisplatin, and 76.4% and 78.9% for etoposide, but only 28.8% and 35.2% of patients in arm A and arm B completed full two cycles of concurrent chemotherapy. The most common reasons for omitting or lowering the doses of concurrent chemotherapy were neutropenia and esophagitis.

Toxicity of induction chemotherapy was mild, and the most frequent grade 3/4 toxicity was neutropenia equally distributed in both arms. In arm B, one case of peripheral ischemia with consequent amputation of lower limb was observed. In recent years, some reports showed possible toxic vascular effects of gemcitabine.^24^,^25^ Among them thrombotic microangiopathy, venous thrombembolism and acute arterial events (digital ischemia and necrosis, vasculitis) are reported. Vascular events due to gemcitabine seem to be more common in patients with tobacco-associated cancers, as it was also in our case.^24^

Treatment toxicity was more obvious in concurrent radiation therapy and chemotherapy. Grade 3/4 anemia and thrombocytopenia were significantly more common in arm A, whereas alopecia was significantly more common in arm B. Since all patients were treated with the same concurrent chemotherapy, part of the toxicity during radiochemotherapy could be attributed to induction chemotherapy. Alopecia was also recognized as significantly more common toxic effect of low-dose gemcitabine in our previous trial comparing two different schedules of gemcitabine in patients with advanced NSCLC.^9^

Our results with induction low-dose prolonged gemcitabine with median survival of 28.6 months and 3 and 5-year estimated survival of 46.2% and 32.2% are encouraging. Results of meta-analysis based on individual data provided by six randomized trials comparing concurrent and sequental radiochemotherapy in 1205 patients with locally advanced NSCLC demonstrated survival rate of 18.4% at 3 years and 15.1% at 5 years in concurrent group.^6^

In recent years, there were several attempts to improve results of treatment locally advanced NSCLC. Trials with concurrent radiochemotherapy in combination with induction or consolidation chemotherapy using platinum-based combinations report median survival in the range of 13 to 29.5 months^19^–^23^,^26^–^29^, 3-year survival in the range of 13% to 39.8%^22^,^27^ and 5-year survival in the range of 12.5% to 22%.^19^,^26^,^29^

A recent pooled analysis of 41 phase II/III trials has confirmed that there remains no evidence to suggest that consolidation chemotherapy after concurrent radiochemotherapy improves survival for patients with stage III NSCLC.^30^

Other recent reports on the treatment of locally advanced NSCLC included new drugs such as pemetrexed and cetuximab in combination with radiotherapy and also sequentially.

Median survival of 19.4 months was reported for 40 patients treated with cetuximab concurrently with radiotherapy followed by consolidation therapy with carboplatin and paclitaxel.^31^ Among 75 patients, treated with concurrent cetuximab and radiotherapy after docetaxel-cisplatin induction chemotherapy, median survival of 17 months and 3-years OS of 29% were reported.^32^ With pemetrexed and cisplatin concurrently with radiotherapy followed by consolidation docetaxel, 28 patients were treated and median survival of 34 months and 1-year survival of 66% was achieved.^33^ In a randomized phase II trial of 4 cycles of carboplatinpemetrexed and concurrent radiotherapy folowed by pemetrexed without or with addition of cetuximab (101 patients), 18-months OS of 58% in the arm without and 54% with cetuximab and median OS of 21.2 months without and 25.2 months with cetuximab were reported.^34^

Another phase III randomized trial of maintenance gefitinib vs. placebo in patients with stage III NSCLC, unselected for EGFR status, who had responded to concurrent radiochemotherapy and consolidation docetaxel demonstrated worse survival in the gefitinib arm. Median survival of 35 months in the control arm compares favourably with results from other phase III studies, although a selection bias must be stressed as patients were randomized following a response to concurrent radiochemotherapy and consolidation chemotherapy.^35^

The most promising results so far have been achieved with trimodality treatment.^36^,^37^ A multi-center phase II trial (CISTAXOL)^36^ showed long-term survival of induction chemotherapy with three cycles cisplatin/paclitaxel followed by concurrent radiochemotherapy cisplatin/etoposide and surgery in locally advanced NSCLC. The median survival was 25 months with 5 and 10-year survival rates of 30.2% and 26%, respectively. In spite of nearly two thirds of the 64 patients in the trial in stage IIIB, the R0-resection rate was 50%. However, trimodality treatment is suitable only for a subgroup of patients with locally advanced NSCLC. A larger number of patients is required for any meaningful conclusion concerning the selection of patients for trimodality treatment.

In conclusion, treatment of locally advanced unresectable NSCLC has not significantly progressed in the last decade, in spite of major changes and improvement in treatment of advanced NSCLC. Combined concurrent radiation therapy and chemotherapy with cisplatin-based combinations remains the standard of care for patients in good performance status and no major comorbidities. In comparison with radiation therapy alone, concurrent radiochemotherapy improves local control. However, no trial so far has demonstrated any influence of concurrent chemotherapy to reduce the high risk of systemic failure, probably due to relatively low dose of cytotoxic drugs when applied together with radiation therapy. Contrary to widely held view that there is no clear benefit of additional chemotherapy before or after concurrent radiochemotherapy, we do believe that systemic control of the disease is of crucial importance for improvement of long-term prognosis. Further trials of induction chemotherapy are therefore warranted, with emphasis on two aspects: individual definition of the optimal schedule of chemotherapy and short gap between completion of chemotherapy and initiation of radiotherapy to avoid repopulation of the tumor cells during this interval.

Our trial compared two cisplatin-based doublets of induction chemotherapy for inoperable NSCLC. Both schedules of gemcitabine had a comparable toxicity profile. Figures for RR, PFS and OS are among the best reported in current literature. In comparison with the standard gemcitabine-cisplatin schedule, the schedule with low-dose gemcitabine in prolonged infusion led to improved long-term survival, but the number of patients is too small for any definitive conclusion. In the future, prognostic and predictive biological and other markers for identify the subgroups of patients for the most optimal schedule of chemotherapy and individualized radiation therapy with isotoxic prescription dose might lead to personalized therapy of patients with inoperable NSCLC.

## Figures and Tables

**TABLE 1.** Characteristics of patients in each treatment arm

	**ARM A (n = 52)** **Standard gem – cis**	**ARM B (n = 54)** **Low-dose gem - cis**	**TOTAL**	**P**
**Gender**				0.42
**Male**	39	44	83	
**Female**	13	10	23	
**Age**				0.41
**Median**	58	57	57	
**Range**	42–72	30–77	30–77	
**ECOG PS**				0.79
**0**	44	47	91	
**1**	8	7	15	
**Weight loss**				0.20
**≤ 5 %**	41	47	88	
**> 5 %**	11	6	17	
**Histology**				0.18
**Squamous-cell carcinoma**	35	27	62	
**Adenocarcinoma**	7	16	23	
**Large cell carcinoma**	4	3	7	
**Other& Unspecified**	6	8	14	
**Stage**				0.15
**IA**	1	0	1	
**IB**	1	0	1	
**IIB**	1	2	3	
**IIIA**	19	31	50	
**IIIB**	30	21	51	
**Reason for inoperability**				0.36
**Extent of disease**	49	52	101	
**Functional**	3	1	4	
**Refuse**	0	1	1	
**Previous treatment**				0.09
**No**	48	44	92	
**Explorative thoracotomy**	3	10	13	
**Recurrent after lobectomy**	1	0	1	

cis = ciplatin; ECOG PS = performance status based on the Eastern Cooperative Oncology Group; gem = gemcitabine; n = number of patients

**TABLE 2.** Treatment delivery

	**ARM A** **No. of patients**	**%**	**ARM B** **No. of patients**	**%**	**P**
**Induction chemotherapy**					
**3 cycles**	28	53.8	24	44.4	0.33
**2 cycles**	23	44.2	27	50.0	0.55
**1 cycle**	1	1.9	3	5.6	0.33
**Concomitant chemotherapy**					
**2 cycles**	15	28.8	19	35.2	0.48
**1 cycle**	26	50	24	44.4	0.57
**Incomplete 1 cycle**	10	19.1	9	16.7	0.92
**No chemotherapy**	1	1.9	2	3.7	0.39
**Radical radiotherapy**					
**Started radical RT**	50	100	53	98.1	0.54
**Surgical treatment after induction chemotherapy**	0	0	1 (pulmectomy)	1.9	0.32
**RT doses >60 Gy**	44	84.6	47	87.0	0.72
**RT doses >56 Gy**	48	92.4	53	98.1	0.16

**PARAMETERS OF RADIOTHERAPY**	**ARM A**		**ARM B**		

**GTV (cm^3^)**					
**Median**	123		124		0.98
**Range**	12–381		11–658		
**PTV (cm^3^)**					
**Median**	619		626		0.99
**Range**	133–1282		210–1428		
**V5 (%)**					
**Median**	64		61		0.52
**Range**	21–88		27–86		
**V20 (%)**					
**Median**	33		33		0.46
**Range**	12–56		20–52		
**V20 > 40%**					
**No. of patients**	12	24	6	12	0.12
**MLD (Gy)**					
**Median**	20		19		0.34
**Range**	6–30		12–27		
**V50oes (%)**					
**Median**	47		42		0.06
**Range**	15–82		2–68		
**MoesD (Gy)**					
**Median**	35		32		0.17
**Range**	7–52		11–44		

GTV = gross tumor volume; MoesD = mean esophagus dose in Gy; MLD = mean lung dose in Gy; No. = number; PTV = panning target volume; RT = radiotherapy; V5 = volume of lung receiving at least 5 Gy; V20 = volume of lung receiving at least 20 Gy; V50oes = volume of esophagus receiving at least 50 Gy

**TABLE 3.** Toxicity of induction chemotherapy

	**ARM A (n = 52)**				**ARM B (n = 54)**				P^*^
	
	**Grade 1, 2**		Grade^*^ 3,4		**Grade 1, 2**		Grade^*^ 3,4		
	
	**No.**	**(%)**	**No.**	**(%)**	**No.**	**(%)**	**No.**	**(%)**	
**Anemia**	47	90.4	**1**	1.9	50	92.6	**0**	0	0.31
**Neutropenia**	11	21.2	**14**	26.9	13	24.1	**11**	20.4	0.43
**Thrombocytopenia**	15	28.8	**0**	0	10	18.5	**1**	1.9	0.32
**Acute kidney injury**	16	30.8	**0**	0	19	35.2	**0**	0	/
**Nausea/vomiting**	19	36.5	**1**	1.9	20	37.0	**3**	5.6	0.33
**Peripheral ischemia**	0	0	**0**	0	0	0	**1**	1.9	0.32
	Grade 1		**Grade 2**		Grade 1		**Grade 2**		
**Alopecia**	11	21.1	**8**	15.4	7	13.0	**23**	42.6	**0.002**

n = number of patients; No. = number;

* statistical significance for grade 3, 4

**TABLE 4.** Hematological and non-hematological toxicity of concurrent radiation and chemotherapy

	**ARM A (N = 52)**				**ARM B (N = 54)**				P^*^
	
	**Grade 1, 2**		Grade^*^ 3, 4		**Grade 1, 2**		Grade^*^ 3, 4		
	
	**No.**	**(%)**	**No.**	**(%)**	**No.**	**(%)**	**No.**	**(%)**	
**Anemia**	48	92.3	**4**	7.7	53	98.1	**0**	0	0.04
**Neutropenia**	17	32.7	**15**	28.8	13	24.1	**14**	25.9	0.74
**Febrile neutropenia**	/	/	**4**	7.7	/	/	**2**	3.7	0.37
**Sepsis**	/	/	**2**	3.8	/	/	**0**	0	0.15
**Pneumonia**	0	0	**2**	3.8	0	0	**0**	0	0.15
**Thrombocytopenia**	24	46.1	**5**	9.6	27	50.0	**0**	0	0.02
**Acute kidney injury**	21	40.4	**1**	0	20	37.0	**0**	0	0.31
**Pericarditis**	0	0	**1**	1.9	0	0	**0**	0	0.31
**Nausea/vomiting**	6	11.5	**3**	5.8	10	18.5	**4**	7.4	0.73
**Esophagitis**	30	57.7	**9**	17.3	39	72.2	**6**	11.1	0.36
**Pneumonitis**	1	1.9	**4**	7.7	4	7.4	**1**	1.8	0.16
**Neurotoxicity**	6	11.5	**0**	0	13	24.1	**0**	0	/
**Weight loss**									
**No**			**35**				**36**		0.94
**1–5%**			**7**				**6**		0.71
**6–20%**			**8**				**8**		0.93
**21–31%**			**0**				**2**		0.16

n = number of patients; No. = number;

* statistical significance for grade 3, 4

**TABLE 5.** Summary of response rates by treatment arm

		**ARM A No. of patients**	**%**	**ARM B No. of patients**	**%**	**P**
**Response rate after induction chemotherapy-RECIST**	CR	0	0	0	0	/
	PR	25	48.1	31	57.4	0.34
	SD	27	51.9	22	40.7	0.25
	PD	0	0	1	1.9	0.32
**Response rate after induction chemotherapy-volumetric results**	V (cm^3^) (median) before ChT	145		124		0.55
	V (cm^3^) (median) after ChT	40.9		28.2		0.26
	Reduction (median, %)	62.6		64.7		0.41
**Response 5 months after completion of treatment**	CR	18		14		0.33
	PR	14		19		0.36
	SD	1		5		0.10
	PD	5		6		0.80
**Median PFS (month)**		15.7		18.9		0.24
**Median OS (month)**		24.8		28.6		0.18
**1-year OS (%)**		38	73.1	44	81.5	0.30
**2-year OS (%)**		27	51.9	30	55.6	0.71
**3-year OS (%)**		17	32.7	24	46.2	0.15
**4-year OS (%)**		11	24.2	17	38.0	0.43
**5-year OS (%)**		7	19.1	11	32.2	0.22
**Site of the first relapse**	No relapse	11	21.2	11	20.4	0.74
	Locoregional	18	48.6	21	53.9	0.51
	Distant	12	32.5	10	25.6	0.56
	Both	7	18.9	8	20.5	0.84
	CNS as the first site of relapse	9	24.3	8	20.5	0.73

ChT = chemotherapy; CNS = central nervous system; CR = complete response; No. = number; OS = overall survival; PD = progressive disease; PFS = progression-free survival; PR = partial response; RECIST = response evaluation criteria in solid tumor; SD = stable disease; V = volume

## References

[1] Ferlay J, Steliarova-Foucher E, Lortet-Tieulent J, Rosso S, Coebergh JWW, Comber H (2013). Cancer incidence and mortality patterns in Europe: estimates for 40 countries in 2012. Eur J Cancer.

[2] Siegel R, Naishadham D, Jemal A (2012). Cancer statistics. CA Cancer J Clin.

[3] Pfister DG, Johnson DH, Azzoli CG, Sause W, Smith TJ, Baker S (2004). American Society of Clinical Oncology treatment of unresectable non-small cell lung cancer guideline: update 2003. J Clin Oncol.

[4] O′Rourke N, Roque i Figuls M, Farre Bernardo N, Macbeth F (2010). Concurrent chemoradiotherapy in non-small cell lung cancer. Cohrane Database Systematic Reviews.

[5] Curran WJ, Paulus R, Langer CJ, Komaki R, Lee JS, Hauser S (2012). Sequential vs. concurrent chemoradiation for stage III non-small cell lung cancer: randomized phase III trial RTOG 9410. J Natl Cancer Inst.

[6] Aupérin A, Le Péchoux C, Rolland E, Curran WJ, Furuse K, Fournel P (2010). Meta-analysis of concomitant versus sequential radiochemotherapy in locally advanced non-small-cell lung cancer. J Clin Oncol.

[7] Kovac V, Smrdel U (2004). Meta-analyses of clinical trials in patients with non-small cell lung cancer. Minireview. Neoplasma.

[8] Rezonja R, Knez L, Cufer T, Mrhar A (2013). Oral treatment with etoposide in small cell lung cancer - dilemmas and solutions. Radiol Oncol.

[9] Zwitter M, Kovac V, Smrdel U, Vrankar M, Zadnik V (2009). Gemcitabine in brief versus prolonged low-dose infusion, both combined with cisplatin, for advanced non-small cell lung cancer: a randomized phase II clinical trial. J Thorac Oncol.

[10] Zwitter M, Kovac V, Rajer M, Vrankar M, Smrdel U (2010). Two schedules of chemotherapy for patients with non-small cell lung cancer in poor performance status: a phase II randomized trial. Anticancer Drugs.

[11] Fossela FV, Lipman SM, Shin DM, Tarassoff P, Calayag-Jung M, Perez-Soler R (1997). Maximum-tolerated dose defined for single-agent gemcitabine: a phase I dose-escalation study in chemotherapy-naive patients with advanced non-small-cell lung cancer. J Clin Oncol.

[12] Touroutoglou N, Gravel D, Raber MN, Plunkett W, Abbruzzese JL (1998). Clinical results of a pharmacodynamically-based strategy for higher dosing of gemcitabine in patients with solid tumors. Ann Oncol.

[13] Anderson H, Thacher N, Walling J, Hansen H (1996). A phase I study of a 24 hour infusion of gemcitabine in previously untreated patients with inoperable non-small-cell lung cancer. Br J Cancer.

[14] Maurel J, Zorrila M, Puertolas T, Antón A, Herrero A, Artal A (2001). Phase I trial of weekly gemcitabine at 3-h infusion in refractory, heavily pretreated advanced solid tumors. Anticancer Drugs.

[15] Pollera CF, Ceribelli A, Crecco M, Oliva C, Calabresi F (1997). Prolonged infusion gemcitabine: a clinical phase I study at low- (300mg/m^2^) and high-dose (875mg/m) levels. Invest New Drugs.

[16] Cattel L, Airoldi M, Delprino L, Passera R, Milla P, Pedani F (2006). Pharmacokinetic evaluation of gemcitabine and 2′,2′-difluorodeoxycytidine-5′-triphosphate after prolonged infusion in patients affected by different solid tumors. Ann Oncol.

[17] Kovac V, Zwitter M, Rajer M, Marin A, Debeljak A, Smrdel U (2012). A phase II trial of low-dose gemcitabine in a prolonged infusion and cisplatin for malignant pleural mesothelioma. Anticancer Drugs.

[18] Zwitter M, Kovac V, Smrdel U, Kocijancic I, Segedin B, Vrankar M (2005). Phase I-II trial of low-dose gemcitabine in prolonged infusion and cisplatin for advanced non-small cell lung cancer. Anticancer Drugs.

[19] Schallier D, Bral S, Ilsen B, Neyns B, Fontaine C, Decoster L (2009). Final overall results of a study with a novel triplet induction chemotherapy regimen (PACCAGE) followed by consolidation radiotherapy in locally advanced inoperable non-small cell lung cancer (NSCLC). J Thorac Oncol.

[20] Hirsh V, Soulieres D, Duclos M, Faria S, Dell Vecchio P, Ofiara L (2007). Phase II multicenter trial with carboplatin and gemcitabine induction chemotherapy followed by radiotherapy concomitantly with low-dose paclitaxel and gemcitabine for Stage IIIA and IIIB non-small cell lung cancer. J Thorac Oncol.

[21] Krzakowski M, Provencio M, Utracka-Hutka B, Villa E, Codes M, Kuten A (2008). Oral vinorelbine and cisplatin as induction chemotherapy and concomitant chemo-radiotherapy in stage III non-small cell lung cancer: final results of an international phase II trial. J Thorac Oncol.

[22] Leong SS, Fong KW, Lim WT, Toh CK, Yap SP, Hee SW (2010). A phase II trial of induction gemcitabine and vinorelbine followed by concurrent vinorelbine and radiotherapy in locally advanced non-small cell lung cancer. Lung Cancer.

[23] Descourt R, Vergnenegre A, Barlesi F, Lena H, Fournel P, Falchero L (2011). Oral vinorelbine and cisplatin with concurrent radiotherapy after induction chemotherapy with cisplatin and docetaxel for patients with locally advanced non-small cell lung cancer: the GFPC 05-03 study. J Thorac Oncol.

[24] Grasic Kuhar C, Mesti T, Zakotnik B (2010). Digital ischemic events related to gemcitabine: report of two cases and a systematic review. Radiol Oncol.

[25] Holstein A, Batge R, Egberts EH (2010). Gemcitabine induced digital ischemia and necrosis. Eur J Cancer Care (Engl).

[26] Curran WJ, Paulus R, Langer CR, Komaki R, Lee JS, Hauser S (2011). Sequential vs concurrent chemoradiation for stage III non-small cell lung cancer: randomized phase III trial RTOG 9410. J Natl Cancer Inst.

[27] Berghmans T, Van Houtte P, Paesmans M, Giner V, Lecomte J, Koumakis G (2009). A phase III randomized sstudy comparing concomitant radiochemotherapy as induction versus consolidation treatment in patients with local-lyy advanced unresectable non-small cell lung cancer. Lung Cancer.

[28] Senan S, Cardenal F, Vansteenkiste J, Stigt J, Akyol F, De Neve W (2011). A randomized phase II study comparing induction or consolidation chemotherapy with cisplatin-docetaxel, plus radical concurrent chemoradiotherapy with cisplatin-docetaxel, in patients with unresectable locally advanced non-small-cell lung cancer. Ann Oncol.

[29] Garrido P, Rosell R, Arellano A, Andreu F, Domine M, Perez-Casas A (2013). Randomized phase II trial of non-platinum iduction or consolidation chemotherapy plus concomitant chemoradiation in stage III NSCLC patients: mature results of the Spanish Lung Cancer Group 0008 study. Lung Cancer.

[30] Tsujino K, Kurata T, Yamamoto S, Kawaguchi T, Kubo A, Isa S (2013). Is consolidation chemotherapy after concurrent chemo-radiotherapy beneficial for patients with locally advanced non-small-cell lung cancer? A pooled analysis of the literture. J Thorac Oncol.

[31] Ramalingam SS, Kotsakis A, Tarhini AA, Heron DE, Smith R, Friedland D (2013). A multicenter phase II study of cetuximab in combinaton with chest radio-therapy and consolidation chemotherapy in patients with stage III non-small cell lung cancer. Lung Cancer.

[32] Hallquist A, Wagenius G, Rylander H, Brodin O, Holmberg E, Loden B (2011). Concurrent cetuximab and radiotherapy after docetaxel-cisplatin induction chemotherapy in stage III NSCLC: satellite-a phase II study from the Swedish Lung Cancer Study Group. Lung Cancer.

[33] Gadgeel SM, Ruckdeschel JC, Patel BB, Wozniak A, Konski A, Valdivieso M (2011). Phase II study of pemetrexed and cispaltin, with chest radiotherapy followed by docetaxel in patients with stage III non-small cell lung cancer. J Thorac Oncol.

[34] Govindan R, Bogart J, Stinchcombe T, Wang X, Hodgson L, Kratzke R (2011). Randomized phase II study of pemetrexed, carboplatin, and thoracic radiation with or without cetuximab in patients with locally advanced unresectable non-small-cell lung cancer: Cancer and Leukemia Group B Trial 30407. J Clin Oncol.

[35] Kelly K, Chansky K, Gaspar LE, Albain KS, Jett J, Ung YC (2008). Phase-III trial of maitanance gefitinib or placebo after concurrent chemoradiation and docetaxel consolidation in inoperable stage III non-small cell lung cancer. SWOG S0023. J Clin Oncol.

[36] Eberhardt WEE, Gauler TC, LePechoux, Stamatis G, Bildat S, Krbek T (2013). 10-year long-term survival (LTS) of induction chemotherapy with three cycles cisplatin/paclitaxel followed by concurrent chemoradiation cisplatin/ etoposide/45Gy (1.5Gy bid) plus surgery in locally advanced non-small-cell lung cancer (NSCLC) - a multicenter phase-II trial (CISTAXOL). Lung Cancer.

[37] Albain KS, Swann RS, Rusch VA, Turrisi AT, Shepherd FA, Smith C (2009). Radiotherapy plus chemotherapy with or without surgical resection for stage III non-small-cell lung cancer. Lancet.

